# The Characteristics of 924 Cutaneous Mast Cell Tumours in Dogs ≤ 3 Years of Age—With a Short Literature Review of Feline, Equine, and Human Proliferative Mast Cell Disorders

**DOI:** 10.3390/vetsci13050500

**Published:** 2026-05-20

**Authors:** Joshua Schwinn, Katrin Törner, Christoph Beitzinger, Robert Klopfleisch, Heike Aupperle-Lellbach

**Affiliations:** 1 LABOKLIN GmbH & Co. KG, 97688 Bad Kissingen, Germany; 2 Institute of Veterinary Pathology, Free University of Berlin, 14195 Berlin, Germany

**Keywords:** Boxer, Danish–Swedish farm dog, English Setter, epidemiology, Golden Retriever, grading, Labrador, mast cell tumour, paediatric, Shar Pei

## Abstract

Datasets from 849 dogs ≤ 3 years of age were analysed retrospectively, considering breed, sex, anatomic sites, multiplicity, tumour diameter, grading, and immunohistochemistry by using IBM^®^ SPSS Statistics (version 29.0.2.0). Danish–Swedish farm dogs (OR: 8.11, 95% CI 3.33–19.77, *p* < 0.001) and English Setters (OR: 6.68, 95% CI 3.20–13.97, *p* < 0.001) showed the highest odds ratios (ORs) compared to crossbreeds. German Shepherd Dogs showed lower ORs (OR: 0.05, 95% CI 0.007–0.364, *p* < 0.01). The most common sites affected in the young dogs were the trunk (39.2%), hind limbs (24.9%), head (10.1%), fore limbs (7.4%), and pinnae (5%). Patnaik grading revealed 330 grade I (35.7%), 580 grade II (62.8%), and 14 grade III MCTs (1.5%). Kiupel “low grade” was found in 903 MCTs (97%), and 21 MCTs were “high grade” (3%). The prevalence of grade I MCTs was higher in Boxers (48.0%, *p* < 0.05) and Pugs (67.9%, *p* < 0.01). Pinnal MCTs were mostly grade I (58.3%, *p* < 0.01). In 6% (*n* = 51), multilocalised MCTs were found, and Golden Retrievers (12.5%, *p* = 0.02) and Pugs (21.4%, *p* = 0.005) were more affected than crossbreeds. Out of 87 dogs, 20 showed a Ki-67 count above the cut-off as well as KIT staining pattern II. The findings warrant heightened vigilance in evaluating cutaneous lesions in young dogs and highlight similarities and differences compared to the general dog population. Analysis of survival data is required to evaluate how the findings translate into a clinical setting.

## Simple Summary

Mast cell tumours are the most common round cell tumours in the skin of dogs. In young dogs, benign histiocytomas occur very frequently, but potentially malignant mast cell tumours are an important differential diagnosis. Our study aimed to characterise the cutaneous mast cell tumours in dogs ≤ 3 years in more detail to improve clinical and diagnostic differentiation. The study retrospectively analysed 924 mast cell tumours submitted from 849 dogs for routine diagnostics between 2014 and 2023. Our data suggest that most breeds with a well-known breed predisposition to cutaneous mast cell tumours have a high risk at a young age. Overall, the proportion of high-grade mast cell tumours was lower compared to older dog populations. However, significant breed-related differences in the prevalence of high-grade tumours were identified: Boxers and Pugs developed low-grade mast cell tumours more often than crossbreeds, and tumours on the ear were more often low grade. Mast cell tumours developed in similar locations to those reported in adult dogs, most commonly involving the trunk. Pugs and Golden Retrievers more frequently showed multiple mast cell tumours simultaneously. Our findings warrant heightened vigilance in evaluating cutaneous lesions in young dogs and highlight similarities and differences compared with the general dog population.

## 1. Introduction

Mast cell tumours (MCTs) have been described in various species, predominantly in dogs, humans, cats, and horses. However, notable species-specific differences in mast cell proliferative disorders have been described.

In dogs, MCTs are usually solitary and, less frequently, multiple. Cutaneous MCTs may appear anywhere on the body, but most commonly arise on the trunk and hind limbs [1,2,3]. Multiple primary cutaneous MCTs are commonly encountered on the trunk in Pugs and often have a favourable prognosis [4]. Perioral and oral MCTs have been associated with a worse prognosis compared to cutaneous manifestations [5]. The prognosis of cutaneous inguinal and perineal MCTs has been thought to be worse than that of other cutaneous sites. However, recent analysis produced no evidence to support this assumption [6,7,8]. The visceral organs are less frequently affected compared to the skin, and there is a predilection for the small intestine. Prognosis is often poor [9,10]. Reports on the effect of sex/castration on MCT risk are adverse, with some data stating an increased risk for spayed bitches [2,11], while others report a lower risk for neutered/spayed compared to intact dogs [12].

The overall peak prevalence for cutaneous MCTs is between eight and nine years of age [12]; however, it may be lower for certain breeds like Shar Peis [13]. The risk for MCTs in young dogs may vary significantly, and especially Shar Peis are known for early onset and higher prevalence of grade III MCTs [1,13]. Multiple breeds with an increased risk for development of MCTs have been identified, including Labrador and Golden Retrievers, Boxers, Pug Dogs, French Bulldogs, Rhodesian Ridgebacks, as well as Staffordshire Bull Terrier and Weimaraners, among others [14,15]. Boxers, Labrador Retrievers, and Golden Retrievers are repeatedly mentioned in studies on MCTs in young dogs [1,16,17]. Statistical analysis providing factual data on risk factors and clinicopathological aspects, specifically in young dogs, remains lacking.

The diagnosis and prognostication in canine cutaneous MCTs rely on histological grading. MCTs are graded according to Patnaik et al. (grades I, II, and III) [18] and/or Kiupel et al. (grades “low/high grade”) [19]. Patnaik grade I and Kiupel “low-grade” MCTs have a good prognosis after surgical excision [19,20]. Cutaneous MCTs grade III and “high-grade” MCTs are associated with shorter survival times and higher rates of local recurrence [19]. The outcome of subcutaneous MCTs (SCMCTs) is usually more favourable compared to cutaneous MCTs if excised completely [21]. Established grading systems do not apply to SCMCTs. However, a recent study suggested that the two-tier grading according to Kiupel might be useful in the detection of biologically aggressive SCMCTs [22]. Immunohistochemical staining for the KIT protein and the Ki-67 antigen can be performed on cutaneous MCTs to refine prognosis. Cytoplasmic location (staining pattern III) of immunohistochemically stained KIT was significantly associated with a higher risk of local recurrence and reduced survival rate [23,24]. The overall survival time was significantly lower in dogs with a high number of Ki-67-positive cells [25], which was pronounced in incompletely excised MCTs [26]. MCTs with histologic grade III are more likely to carry a mutation in the *kit* gene [27,28]. The internal tandem duplication (ITD) in Exon 11 of the *kit* gene was associated with shorter survival [29,30]. A variable number of canine cutaneous MCTs do not show a mutation in *kit* [27,31,32]. MCTs harbouring a mutation in *kit* are more sensitive to tyrosine-kinase inhibitors [33].

Compared to dogs, cutaneous MCTs are less common in cats. They make up to 16% of tumours in cats ≤ 1 year of age [34,35]. Visceral MCTs are more frequently observed in cats than in dogs: up to 50% of cats with MCTs have visceral involvement [36,37]. Feline cutaneous MCTs are commonly found on the head and neck, whereas visceral MCTs predominantly involve the spleen [38]. Siamese cats < 4 years of age may be at higher risk for developing “atypical” cutaneous MCTs (formerly termed “histiocytic”) [37]. Multiple cutaneous MCTs occur in up to 20% of affected cats [39]. In contrast to dogs, cats with multiple MCTs have a worse prognosis than solitary MCTs [39,40]. An analysis of 61 feline MCTs found that only about 60% of cutaneous MCTs were immunohistochemically positive for KIT. Ki-67 count is a reliable indicator of high-grade malignancy and a worse prognosis in cats with MCTs [39]. Furthermore, the grading system for cutaneous MCTs in felines proposed by Sabattini et al. includes mitotic count (MC), tumour diameter, and nuclear atypia as prognostic parameters for MCT [39]. Splenic MCTs with mutated *kit* in Exon 8 have been reported. Furthermore, in vitro sensitivity to tyrosine kinase inhibitors (TKIs) has been documented [41,42].

Horses rarely develop cutaneous MCTs, with only 19 out of 536 cutaneous neoplasms being mast cell tumours in a USA-based retrospective analysis of a Veterinary Diagnostic Laboratory over the span of 3.5 years [43]. The most frequent sites affected are the trunk and head [43,44,45]. The large majority of mast cell tumours in horses have a favourable prognosis [45]; however, a malignant course of disease has been reported [46]. The prevalence and prognosis of MCTs in young horses are not known, but a case of congenital cutaneous mastocytosis with a progressive clinical course has been described in a foal [47].

In humans, mast cell tumour disease is classified into cutaneous mastocytosis (CM) and systemic mastocytosis (SM) [48]. Based on clinical presentation, the umbrella term cutaneous mastocytosis encompasses maculopapular cutaneous mastocytosis, urticaria pigmentosa, diffuse mastocytosis, and lastly cutaneous mastocytoma with isolated and multilocalised presentation [49]. Cutaneous involvement in adults more frequently develops secondary to underlying SM and a major diagnostic criterion is the presence of mast cells in bone marrow and/or other extracutaneous organs [50]. Children affected by CM predominantly show polymorphic maculopapular mastocytosis [51,52]. In contrast, a solitary mastocytoma more frequently appears perinatally, developing before the age of two years, or is congenital. Lesions usually resolve spontaneously, however, in rare cases, they may persist [53]. Solitary non-cutaneous mastocytomas are very rare and usually benign. Of the few tumours reported, all involved the lungs [54]. Reports on gender predisposition are conflicting, with some studies identifying women [55] and others men [50,56] as overrepresented.

In humans, the use of KIT and tryptase markers in immunohistochemistry is recommended to facilitate the detection of mast cells in cutaneous lesions [49]. The diagnostic work-up of mast cell disorders in humans always includes the determination of *kit* mutation status, and a D816V mutation on Exon 17 of the *kit* gene is present in the majority of cases with SM [57]. Immunohistochemical detection of CD5, CD20, and CD25 in bone marrow [58] and the detection of the *kit* D816V mutation or other activating *kit* mutations in bone marrow or other extracutaneous organs are minor criteria for diagnosis of SM [51]. In paediatric-onset solitary mastocytoma other *kit* mutations outside Exon 17 may be present [58], and a subset of childhood-onset mastocytosis may not harbour any detectable mutations [59].

Mast cell tumours in dogs differ from human, feline, and equine mast cell neoplasms in various aspects. In contrast to the extensive body of research that has examined various risk factors and clinicopathological aspects of humans (e.g., patient age), an age-focused approach to understanding MCT biology in dogs has not yet been undertaken. MCTs have been diagnosed in dogs as young as two months of age [16] and they are the most common malignant tumour arising in the skin of young dogs [17,34,35]. Despite this, factual data on clinicopathological aspects and the prognosis of cutaneous MCTs in young dogs are scarce, and data on studies for this specific age group are limited.

This study retrospectively describes and statistically compares the features of mast cell tumours within a cohort of young dogs and provides epidemiological data that address the current data gap in the literature. For perspective, the data are embedded in a comparative context of proliferative mast cell disorders in felines, equines, and humans that highlight the current clinical and epidemiological knowledge in other species.

## 2. Materials and Methods

### 2.1. Data Collection

The datasets (*n* = 31,087) were extracted from the custom-made database of LABOKLIN GmbH & Co. KG (Bad Kissingen, Germany) for the years 2014 to 2023, including breed, sex, age, gross pathology, histologic description, diagnosis, and, where available, immunohistochemical results. The patient signalment (breed, sex, and age) was assumed to be accurate. Age was categorised into yearly intervals. Dogs aged ≤ 1 year were assigned to the 1-year category, those aged > 1 to ≤2 years to the 2-year category, and those aged > 2 to ≤3 years to the 3-year category.

Breeds were standardised and summarised according to the latest FCI list (www.fci.be/de/, accessed on 2 February 2026).

General inclusion criteria were as follows:

1. Tissue samples were submitted for histopathological evaluation.
2. Complete patient signalment (age, breed, and sex) was available.
3. The patient was ≤3 years of age at the time of submission.
4. A definitive diagnosis was rendered.

The decision to include dogs ≤ 3 years was made based on the low relative prevalence of neoplasia compared to other age groups. The authors are not aware of a universal biologically meaningful cut-off age for dogs, and determining such a threshold is beyond the scope of the present study. Categorisation and summarisation of diagnoses were based on the International Classification of Diseases for Oncology, Third Edition, First Revision (ICD-O-3, 28 April 2020; WHO).

### 2.2. Variables and Definitions

Histological diagnosis was made by a trained veterinary pathologist and graded according to Patnaik et al. [18] and Kiupel et al. [19] during routine diagnostics. If not otherwise mentioned, the term “grade” refers to Patnaik grades. Throughout the manuscript, the term MCTs refers exclusively to cutaneous mast cell tumours. Subcutaneous, visceral, and muco-cutaneous MCTs were excluded from the analysis.

The sampling sites were derived from submission forms and categorised according to the guidelines published in the supplement to the latest Vet-ICD-o-Canine-1 [60]:

1. Digit_NOS: Including MCTs found on the digits.
2. Head: Including MCTs found on the head, excluding the conjunctivae, nasal planum, and labia, as they were interpreted as “muco-cutaneous MCTs”, and excluding MCTs located on the pinnae.
3. Hind/fore limb: Including MCTs found in the inguinal area and the axillary region, respectively. MCTs appearing on the digits were included in the digit_NOS category.
4. Neck: Including all MCTs located in the skin between the cranial aspect of the shoulder and the base of the skull and pinnae.
5. Pinna: Including MCTs found on the pinnae.
6. Scrotum: Including MCTs located on the scrotum.
7. Trunk: Including MCTs found on the tail.

Tumour size was measured three-dimensionally and was taken from the trimming protocols. To enhance the comparability of results, tumour size was compared using the equivalent spherical diameter, defined as the diameter of a sphere with the same volume as the tumour. Tumour volume was calculated using the volume for a rotational ellipsoid (V = 4/3 × π × a × b × c). The volume of the rotational ellipsoid was inserted into the formula for a sphere, rearranged to solve for the radius (*r* = (3V/4π)^(1/3)^), and the diameter was calculated as “2 × *r*”.

The submissions of multiple MCTs per patient (≥2 MCTs/patient) were interpreted as “multilocalised” MCTs. Only cases with involvement of multiple cutaneous sites were included in the category of “multilocalised” MCTs.

Immunohistochemical staining for KIT (Dako™ Agilent A4502, Dako North America, Inc., Carpinteria, CA 93013, USA; 1:150) and Ki-67 antigen (Dako™ Agilent M7240; Dako North America, Inc., Carpinteria, CA 93013, USA; 1:200) was performed in 87 cases upon the client’s request and validated by a veterinary pathologist. The KIT expression was documented according to Kiupel et al. (2004) and sorted into one of three staining patterns—pattern I: membranous, pattern II: perinuclear, or pattern III: diffuse cytoplasmic [23,61]. The Ki-67 count was calculated as the sum of Ki-67-positive mast cells within an ocular grid (10 × 10 mm) and binomially sorted into categories ≤ 23 or >23 Ki-67-positive mast cells [61].

### 2.3. Composition of Subgroups

For the analysis of specific variables, statistical evaluations were performed in predefined study subsets, as outlined below:

(a) Regression model cohort (*n* = 31,087), comprising histopathological cases from dogs aged ≤ 3 years, corrected for duplicate entries and analysed on the patient level. Within this cohort, the following subgroups were defined:
(b) MCTs_total group (*n* = 924), tumour level, including all cutaneous mast cell tumours diagnosed within the regression model cohort. Datasets were multiplied by the number of MCTs diagnosed in a single patient during a single submission.
(c) MCTs_patient group (*n* = 849), patient level, consisting of all dogs diagnosed with cutaneous MCTs, with one tumour per patient included in the analysis. If more than one MCT was diagnosed in a patient, the highest-grade tumour was included in patient-level analyses.
(d) Sampling-site group (*n* = 715), patient level, comprising dogs affected with cutaneous MCTs with a documented anatomic site. Corrected for duplicate entries.
(e) Tumour-size group (*n* = 406), patient level, including dogs with cutaneous MCTs with a documented tumour size. Corrected for duplicate entries.
(f) Ki-67 and KIT group (*n* = 87), consisting of the subset of dogs for which immunohistochemical examination (Ki-67 and KIT staining) was requested and performed.

### 2.4. Statistical Analysis

Statistical tests were performed using IBM SPSS Statistics, version 29.0.2.0, IBM Corporation, Armonk, NY, USA. Unless stated otherwise, differences in MCT characteristics were evaluated relative to crossbreeds, which served as the reference category (e.g., odds ratio (OR) for crossbreeds = 1). Associations between categorical variables were assessed using Pearson’s chi-square test in a 2 × 2 contingency table. Fisher’s exact test was used when expected cell counts were <5. Breed-specific odds ratios for the occurrence of cutaneous MCTs were calculated using multivariable binary logistic regression models with MCT status (MCT vs. no MCT) as the dependent variable and breed, sex, and age as independent variables. For the regression analysis age was coded as a continuous variable. In analyses evaluating associations across age, age was treated as an ordinal categorical variable. The effect of age on breed-specific odds ratios for developing cutaneous MCTs was evaluated by including age and breed as main effects and an age × breed interaction term in a logistic regression model. For both interaction analysis and logistic regression, crossbreeds served as the reference category. Distribution of tumour diameters across multiple groups (histological grades and age) was analysed using the Kruskal–Wallis test, followed by post hoc pairwise comparison using the non-parametric Mann–Whitney U test for non-normally distributed data. Correlations were analysed using Spearman’s rank correlation with tumour diameter as a continuous variable and histological grade as an ordinal categorical variable. Duplicate entries (e.g., recurring MCTs submitted for histology) were counted once, and the first entry was included in the analysis. To avoid clustering effects, analyses of odds ratios, histological grade proportions, anatomical localisation, and tumour diameter were conducted at the patient level. To control for type I error due to multiple testing, *p*-values were adjusted using the Bonferroni correction by multiplying the original *p*-values by the number of comparisons performed. Adjusted *p*-values ≤ 0.05 were considered significant. Levels of statistical significance are indicated by asterisks (* *p* ≤ 0.05, ** *p* ≤ 0.01, and *** *p* ≤ 0.001).

### 2.5. Ethics Statement

Data collection and analysis were conducted in accordance with the General Data Protection Regulation (GDPR) in conjunction with the German Federal Data Protection Act (BDSG). Only anonymised patient data were used. Individual pets and/or owners cannot be identified, and no personal data were published. The authors confirm that there are no ethical concerns regarding the conduct or publication of this study.

## 3. Results

### 3.1. Logistic Regression Analysis of Signalment Data

#### 3.1.1. Breed

**Figure 1:**
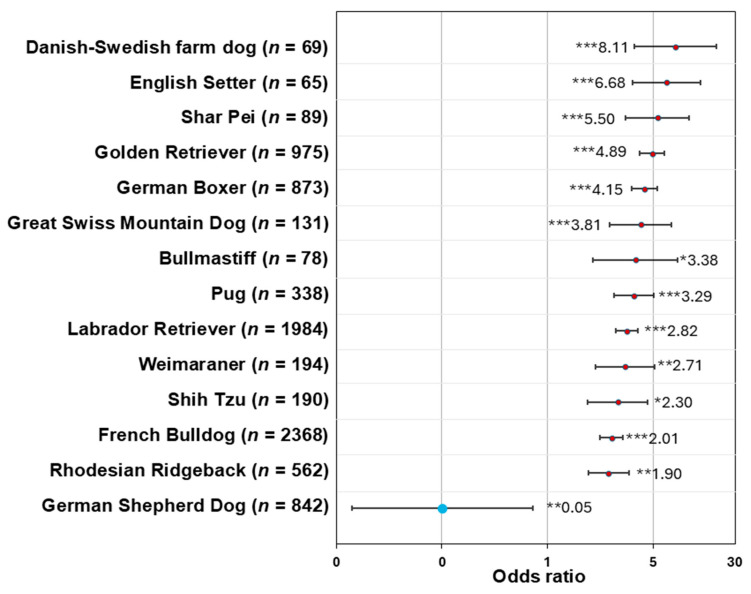
Forest plot showing odds ratios (ORs) for cutaneous mast cell tumours by breed (outcome: cutaneous MCTs/other) in dogs ≤ 3 years of age. ORs were calculated using a multivariable binary regression model and are listed in reference to the odds ratios of crossbreed dogs. The total number of cases (non-neoplastic + neoplastic) in the study cohort for each breed is indicated in parentheses. Horizontal bars represent the 95% confidence interval (CI). Detailed CIs can be found in Table S5. Statistical significance was adjusted for multiple testing (* *p* ≤ 0.05, ** *p* ≤ 0.01, and *** *p* ≤ 0.001).

Included were MCTs of a total of 849 young dogs of various breeds. All analyses were conducted solely on cutaneous MCTs. The majority of dogs were crossbreeds (*n* = 160, 18.9%). The most common pure breed dogs were the Labrador Retriever (*n* = 112, 13.2%), French Bulldog (*n* = 98, 11.5%), and Golden Retriever (*n* = 88, 10.4%). Other common breeds were German Boxer (*n* = 75, 8.8%), Pug (*n* = 28, 3.3%), Rhodesian Ridgeback (*n* = 24, 2.8%), Bernese Mountain Dog (*n* = 15, 1.8%), American Staffordshire Terrier (*n* = 12, 1.4%), Weimaraner (*n* = 11, 1.3%), Great Swiss Mountain Dog (*n* = 10, 1.2%), Shih Tzu (*n* = 10, 1.2%), Boston Terrier (*n* = 5, 0.6%), and Shar Pei (*n* = 10, 1.2%; see Supplementary Table S2 for a full list of breed prevalence). The odds ratios were derived from a multivariable binary logistic regression model with crossbreeds serving as reference breeds and adjusted for sex and age. Danish–Swedish farm dogs (OR: 8.11, 95% CI 3.33–19.77, *p* < 0.001) and English Setters (OR: 6.68, 95% CI 3.20–13.97, *p* < 0.001) showed the highest odds ratio for cutaneous MCTs compared to the odds of crossbreeds in our study cohort. Furthermore, significantly higher odds ratios were found for Labrador Retrievers, Golden Retrievers, French Bulldogs, Rhodesian Ridgebacks, Pugs, German Boxers, and Great Swiss Mountain Dogs (OR 1.89–4.89, *p* < 0.05, Figure 1). In addition, Shar Peis, Bullmastiffs, Weimaraners, and Shih Tzus showed a higher odds ratio than crossbreeds (OR 2.15–6.20, *p* < 0.05, Figure 1). In contrast, the odds ratio was significantly reduced in German Shepherd Dogs (OR: 0.05, 95% CI 0.007–0.364, *p* < 0.01).

#### 3.1.2. Sex

Of the 849 dogs affected by cutaneous MCTs, 415 were males, and 434 were females. Of the male dogs, 28% were neutered (*n* = 117), and of the female dogs, 37% were spayed (*n* = 159). Overall, 32.5% of dogs were neutered or spayed. Compared to intact males, there were no significant differences in odds ratios in our cohort of young dogs.

#### 3.1.3. Age

Of the 849 dogs included in the study, 149 were ≤1 year old, 247 were >1 and ≤2 years old, and 453 were >2 and ≤3 years old. Overall, the MCT risk increased by 1.72 per year of age (*p* < 0.001). Compared to crossbreeds, there were no significant differences in the effect of age on MCT odds for breeds after adjustment for multiple testing.

### 3.2. Histological Grading

Of the 924 cutaneous MCTs, 330 were Patnaik grade I (35.7%), grade II was assigned to 580 MCTs (62.8%), and 14 were grade III MCTs (1.5%). According to Kiupel et al. (2011) [19], 903 cutaneous MCTs were graded as “low-grade” (97%) and 21 as “high-grade” (3%) MCTs.

**Figure 2:**
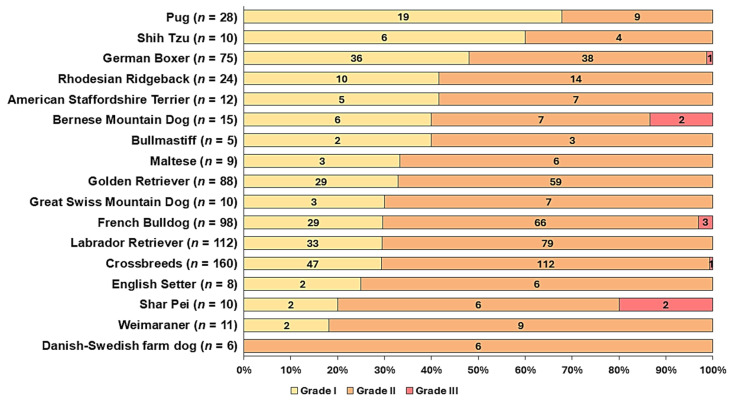
Proportion of Patnaik grades of cutaneous MCTs diagnosed in dogs ≤ 3 years of age. Shown are breeds with a high odds ratio and/or ≥9 dogs included in the cohort. The number in parentheses indicates the total number of dogs in this breed diagnosed with MCTs in the study cohort.

Compared to crossbreeds, the prevalence of grade I MCTs was higher in Boxers (48%, *p* < 0.05) and Pugs (67.9%, *p* < 0.01) than in crossbreeds (see Figure 2). No significance was found for the proportion of grade III MCTs or Kiupel high-grade MCTs in Shar Peis (2/10 grade III/high-grade MCTs, *p* = 0.06) and Bernese Mountain Dogs (2/15 grade III/high-grade MCTs, *p* = 0.14) after adjustment for multiple testing. No association was found between histological grade, age, and sex.

For 10 dogs, the regional lymph node was submitted, and MCT metastases were found in 5. In 4/5 cases the primary MCT was Patnaik grade III and in 1 case it was Patnaik grade II. All MCTs with lymph node metastasis were located on the trunk (see Table S5 for full signalment of affected dogs).

### 3.3. Anatomical Sites

The anatomic site of cutaneous MCTs was known in 715 of 849 dogs. In 39.3% of cases with a known body site, the tumour appeared on the trunk. The hind limbs were affected in 24.9%, and the fore limbs in 7.4%. In 10.1%, MCTs were found on the head, and in 5%, on the pinnae. The neck was affected in 30 dogs (4.2%), and the scrotum and tail were affected in 9 dogs each (both 1.3%). In 11 cases (1.5%), the digit was involved.

The proportion of MCTs on the trunk was significantly lower in one-year-olds (29.4%) compared to two- and three-year-olds (41.4%, *p* = 0.03). French Bulldogs developed more MCTs on the scrotum (6.5%, *p* < 0.05) and fewer on the trunk (31.7%, *p* < 0.05) compared to crossbreeds. The skin of the head was more often affected in Maltese (5/9 Maltese dogs, 55.6%, *p* < 0.01).

**Figure 3:**
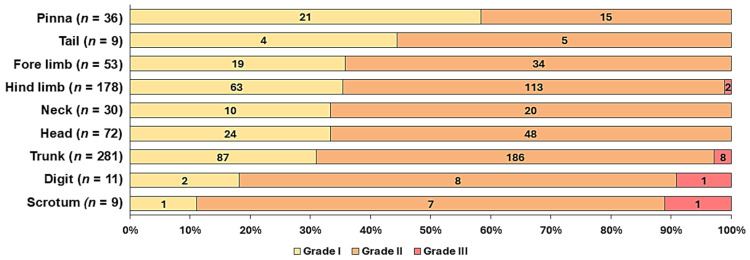
Proportions of histological grades of cutaneous MCTs in dogs ≤ 3 years of age at different anatomic sites. Grading according to Patnaik et al. (1984) [18]. The number in parentheses indicates the total number of dogs in this breed diagnosed with MCTs in the study cohort.

Differences in grade proportions were significant only at the pinna (grade I: 58.3%, *p* < 0.01; Figure 3). The proportion of grade III MCTs in scrotal (*p* = 0.42) and digital (*p* = 0.48) MCTs was not statistically significant. Between sexes, no significant differences in the anatomic distribution of MCTs were observed.

### 3.4. Multiplicity

In a total of 51 dogs (6% of all dogs with MCTs), multilocalised cutaneous MCTs were found. Submissions that included mast cell tumours with lymph node metastasis were not included or interpreted as “multilocalised” MCTs.

**Figure 4:**
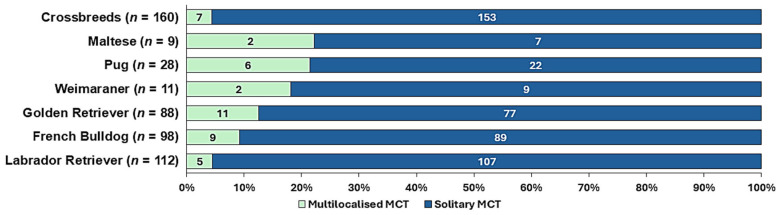
Proportions of multilocalised and solitary cutaneous MCTs in dogs ≤ 3 years of age. The number in parentheses indicates the total number of dogs in this breed diagnosed with MCTs in the study cohort. Recurrent MCTs were not counted as multiple occurrences.

In 39 dogs, 2 MCTs were diagnosed. Six dogs were affected by three MCTs, two were affected by four, and three were affected by five multilocalised MCTs. One Weimaraner presented with six MCTs. Pugs (21.4%, *p* = 0.005) and Golden Retrievers (12.5%, *p* = 0.02) showed a higher prevalence of multiple MCTs than crossbreeds (4.4%) (see Figure 4). A multivariable logistic regression model corrected for sex and age revealed no significantly increased or decreased odds ratios for development of multilocalised MCTs of breeds after adjustment for multiple testing. No association was found between multiplicity, histological grade, or age.

### 3.5. Tumour Size

Tumour size is documented in three dimensions and has been standardised for better comparability. All mentions of tumour diameter and tumour size refer to the equivalent spherical diameter. Tumour size was measured macroscopically and ranged from 1.0 mm to 62.0 mm ($\tilde{x}$ = 9.8 mm). The median tumour diameter in Pugs was significantly smaller ($\tilde{x}$ = 5.0 mm, 2.99–22.10 mm; *p* < 0.01) compared to crossbreeds ($\tilde{x}$ = 9.2 mm, 1.0–35.23 mm). The largest median tumour diameter was recorded for Shar Pei ($\tilde{x}$ = 30.9 mm). The largest tumour diameter recorded was 62.0 mm in a grade III MCT in a three-year-old neutered Bernese Mountain Dog and 50.9 mm in a grade II MCT of a two-year-old intact female Shar Pei.

**Figure 5:**
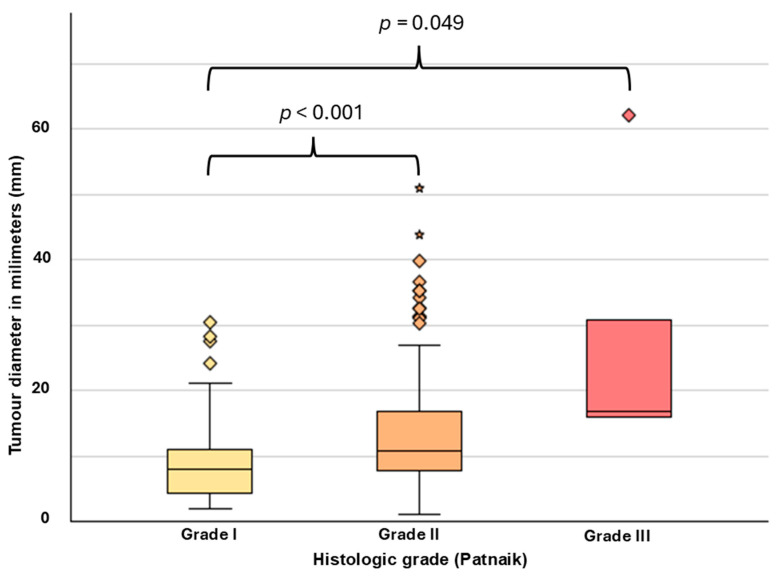
Macroscopic tumour diameters (mm) of cutaneous MCTs in dogs ≤ 3 years of age, grouped by Patnaik grades I–III. *p*-values shown are adjusted for multiple testing using the Bonferroni method.

Between Patnaik grades and tumour diameter, a significant moderate correlation was observed (ρ = 0.326, *p* < 0.001). Between Kiupel grades and tumour diameter, a weak positive correlation was found (ρ = 0.149, *p* < 0.001). Pairwise comparison revealed a significant difference in median tumour diameter between grade I ($\tilde{x}$ = 7.9 mm, 1.97–30.4 mm) and grade II ($\tilde{x}$ = 10.8 mm, 1.0–50.9 mm; *p* < 0.001) and grade I and grade III MCTs ($\tilde{x}$ = 16.8 mm, 15.9–62.1 mm, *p* = 0.049). The difference in median diameter between grade II and III was not significant after adjustment for multiple testing (*p* = 0.08) (see Figure 5). The median tumour diameter difference between solitary and multiple MCTs was not significant (*p* = 0.14). Between median tumour diameters of low grade and high grade, no significant differences were observed (*p* = 0.06).

### 3.6. KIT and Ki-67 Immunohistochemistry

Immunohistochemical analysis (IHC) targeting the Ki-67 and KIT antigens was requested for 87 of the 849 dogs included in the analysis. Labelling for KIT revealed membranous staining of mast cells (staining pattern I) in 42% of cases (*n* = 38), perinuclear staining (staining pattern II) in 55% (*n* = 47), and diffuse cytoplasmic staining (staining pattern III) in 3% (*n* = 2). A borderline association was found between castrated/spayed dogs and KIT staining pattern I (*p* = 0.05). No significant associations were found between KIT staining pattern and age, multiplicity, tumour location, and size.

Of the reports available, 22.9% (*n* = 20) had more than 23 Ki-67-positive cells in a 10 × 10 mm grid area. For 77.1% of dogs (*n* = 67), the Ki-67 count was ≤23 positive (see Table 1). No significant associations were found in the proportion of Ki-67 labelling and grade, sex, age, anatomic site, breed, or tumour size.

## 4. Discussion

This study retrospectively analysed the characteristics of cutaneous mast cell tumours (MCTs) diagnosed in 849 young dogs ≤ 3 years of age.

### 4.1. Limitations

Our study is subject to limitations inherent to retrospective research. The signalment data are based on records submitted by veterinarians, which may be inaccurate or incomplete. The subgroups are selective by definition and do not reflect population prevalence accurately, as full information on anatomic location, tumour diameter, and KIT and Ki-67 immunohistochemistry was not always available. Because clinical outcome data were not included, no comment can be made on the prognostic value of the included variables. As the included datasets were derived solely from tumours submitted to a veterinary diagnostic laboratory for histologic analysis, the scalability and generalisability of the results of our study to the general dog population are limited. The composition of our cohort may be biased and depends on the availability and selection of previously collected samples and records. Due to the small sample size of certain breeds, interpretability may be limited and the results should, therefore, be interpreted with caution.

### 4.2. Logistic Regression Analysis

Breeds associated with an increased risk of cutaneous MCTs in general, such as German Boxer, Golden Retriever, Labrador Retriever, Pug, and French Bulldog [12,62], also showed significantly higher odds in the cohort of young dogs of the present study. Furthermore, in the literature, increased odds have been described for the Weimaraner [63], Rhodesian Ridgeback [15], and Shar Pei [1,13]. A retrospective analysis of specimens submitted to a USA-based diagnostic laboratory reported 17 dogs < 2 years of age affected with MCTs belonging to the breeds Shar Pei, Boxer, Golden Retriever, and Shih Tzu [1]. Golden Retriever, Labrador Retriever, French Bulldog, Pug, Shih Tzu, and Rhodesian Ridgeback were included in an analysis of survival data of nine dogs ≤ 1 year of age affected with MCTs [16]. Our data confirm that these breeds show higher odds for developing cutaneous MCTs, suggesting an increased risk during the first three years of life. Additionally, Irish Setter and Chihuahua were affected breeds; however, increased odds were not found in the data. Notably, the present study further identified the Danish–Swedish farm dog (DSF), the English Setter, and the Shih Tzu as high-risk breeds in young dogs. The Danish–Swedish farm dogs (DSF) showed the highest odds ratio for cutaneous MCTs in this study of young dogs. We found no mention of increased MCT risk or increased odds ratios for this breed in the literature, making this study the first to report it. The odds ratios of DSF and English Setter in our cohort should be interpreted with caution due to the broad confidence intervals, which are probably attributable to the low number of cutaneous MCTs found in these breeds in our cohort.

One study reports an increased MCT risk for “English Setter/Irish Setter”, but odds ratios were expressed relative to all other breeds rather than just crossbreeds [64]. Contrasting the present study, only 3 out of 240 included cases of English Setters (aged 1–16 years) were cutaneous MCTs in another study [2]. However, it is important to note that this cohort comprised dogs submitted to a Veterinary Teaching Hospital [2]. Therefore, dogs may present with different clinical problems and diagnoses, limiting comparability to the present study’s results. English Setters may be at higher risk for development of cutaneous MCTs during the first three years of life. The OR should be interpreted with caution, as broad CIs may suggest underlying uncertainty.

Interestingly, Rigas et al. (2020) [16] reported an Irish Setter with cutaneous MCT (less than 12 months old). In this study’s cohort, 79 Irish Setters ≤ 3 years of age were included, none of whom had been diagnosed with cutaneous or subcutaneous MCTs. Based on the current data, young Irish Setters do not appear to have an increased risk of cutaneous MCTs, despite being genetically related to English Setters.

The only breed showing significantly reduced odds for developing MCTs in young dogs compared to crossbreeds was the German Shepherd Dog (GSD). Of the 849 dogs with cutaneous MCTs in the investigated cohort, none was a GSD. The generally lower risk for cutaneous MCTs of German Shepherd Dogs of all ages has been reported in studies from the US [63,65], Sweden [66], Poland [13], Italy [2], Switzerland [14], and Germany [15]. Similar to the DSF and English Setter, broad CIs were found for the ORs of the GSDs. Due to the consistency of the ORs in the present cohort with the literature, it is likely that the GSDs exhibit a lower risk than crossbreeds for development of cutaneous MCTs during the first three years of life.

Similar to dogs, breed predispositions have been described in cats. An increased risk for feline MCTs has been reported in Siamese, Burmese, Russian Blues, and Ragdolls [67]. Siamese have been mentioned in the context of a mastocytosis-like disease [68]. No study has identified risk factors for MCTs in horses.

The etiopathogenesis of breed-specific predisposition to canine MCTs is unknown. Both germline and somatic mutations have been associated with mast cell tumour disease. A synonymous germline variant of the *DSCAM* gene harbouring cell adhesion molecules has been associated with MCTs in Labrador Retrievers [69]. In Golden Retrievers, a germline single-nucleotide polymorphism (SNP) mutation in the *CNAI2* gene was associated with the development of MCTs [70].

Somatic *kit* mutations have been found in canine MCTs [71]; however, in low-grade MCTs, *kit* mutations are frequently absent [72]. Overall, it has been suggested that *kit* mutations might play a lesser role in the general etiopathogenesis of MCTs in canines [73]. To date, no association between age, breed, and *kit* mutation status has been found in dogs [74], leading to the assumption that different genetic alterations may be at fault. Recent advances in canine genomics may shed further light on the molecular genetic landscape of canine MCTs.

Data about *kit* mutations in young dogs are rare: no mutations in Exons 8 and 11 of the *kit* gene were found in eight young dogs affected with cutaneous mastocytosis [75]. Another study included 11 dogs aged 1–12 years and found an Exon 9 mutation in a 2-year-old Weimaraner and a 4-year-old crossbreed. Mutations in Exon 17 were not found [76]. In our cohort, veterinarians did not order mutation analyses. Thus, no molecular genetic data were available for this retrospective study. However, this could be of interest in further studies.

In humans, the D816V mutation in Exon 17 of *kit* can be found in over 90% of adult systemic mastocytosis (SM) [57] and 40% of paediatric cutaneous mastocytosis (CM) [77]. The D816V *kit* mutation is present in both SM and CM patients [50]. Furthermore, it is important to note that in humans, a germline *kit* mutation in Exon 8 has been described as the ontogenetic driver of familial mastocytosis and gastrointestinal stromal tumours (GISTs) [78]. The difference in clinical presentation of mastocytosis in paediatric onset is thought to be caused by other mutations outside of Exon 17 [79]. According to a recent Swedish study, adults are more frequently affected by mast cell disorders, and the average age of diagnosis was 50.6 years [55].

In the present study, several breeds with an increased odds ratio of cutaneous MCTs were identified. These findings may suggest a higher risk for MCTs early in life and increased vigilance may be advisable when evaluating cutaneous lesions in these breeds.

### 4.3. Histological Grading

Cutaneous MCTs have been described as common neoplasms in dogs < 1 year; however, the prevalence of histologic grades was not reported [17,34,35]. In young cats, a higher prevalence of atypical MCTs (termed “histiocytic” in the original publication) has been reported, and Siamese cats may be predisposed [80]. The prognosis of atypical MCTs is controversially discussed [39]. Regarding the prevalence of cutaneous MCTs in young cats, there are no data in the literature—the overall prevalence and prevalence of high-grade MCTs may be lower than in older cats [39,67,81].

In the present study, 330 Patnaik grade I (35.7%), 580 Patnaik grade II (62.8%), and 14 Patnaik grade III MCTs (1.5%) were diagnosed. The proportion of grade III MCTs in these young dogs was lower than in dogs of all ages: 2.9% were grade III MCTs in an Italian study [2], 8.2% grade III were reported in a US-based study (9375 MCTs included) [65], and 8.3% grade III were found in dogs from Germany (6861 MCTs included) [15]. A Polish study found that dogs aged 11–16 years were more likely to develop Kiupel high-grade MCTs than dogs aged 1–3 years [62]. These findings support the hypothesis that, in general, fewer grade III/high-grade MCTs appear in young dogs. Whether this means a favourable outcome for cutaneous MCTs in young dogs requires follow-up data, which were not available in the present study.

Significant differences in grade proportions were found among breeds: Boxers and Pugs showed significantly higher proportions of grade I MCTs than crossbreeds, which is also well-known from studies including dogs of all ages [4,62]. However, whether MCTs in these breeds truly have a more favourable outcome remains unclear, as survival times or progression-free intervals (PFIs) were unavailable.

Shar Peis, on the other hand, have repeatedly been associated with a greater prevalence of grade III/high-grade MCTs [1,15]. However, in the present study, 2/10 cutaneous MCTs were grade III/high grade, and no significant differences were found. Smiech et al. (2018) also found that Shih Tzus and Bernese Mountain Dogs (BMDs) were at higher risk for high-grade MCTs [62]. While no grade III MCTs were found in the young Shih Tzus in the present study, 2/15 young BMDs with cutaneous MCTs were grade III. Based on our findings, there were no significant differences in grade proportions of BMDs and Shar Peis and further analysis will be required to validate whether these breeds show increased proportions of highly malignant MCTs at a younger age. Inclusion of survival data may be useful to evaluate whether the findings on histologic grades translate into a clinical setting. Molecular genetic analysis of the exon 11 mutation and immunohistochemical characterisation in these breeds could be of particular interest in future studies.

### 4.4. Anatomic Sites

In general, the anatomic site of mast cell tumours is relevant across species. Cutaneous mastocytosis (CM) in children predominantly manifests as polymorphic maculopapular skin lesions on the head, trunk, and extremities, with a good prognosis and spontaneous remission [51]. Adult-onset solitary mastocytoma is rare, and lesions are usually located on the neck, trunk, and extremities. The prognosis is favourable after surgical extirpation [82]. In cats, the skin of the head and neck is more frequently affected; however, splenic MCTs are more common than cutaneous MCTs in general [38]. Horses more frequently develop MCTs on the head, neck, or trunk [45].

The anatomic site of cutaneous MCTs was known in 715 young dogs of the present study. MCTs were found on the trunk (39.2% of cases), hind limbs (24.9%), fore limbs (7.4%, *n* = 53), head (10.1%, *n* = 72), pinna (5%, *n* = 36), neck (4.2%, *n* = 30), digit (1.5%, *n* = 11), and scrotum and tail (9 dogs each, both 1.3%).

Across multiple studies, either the trunk [1,2,62] or the hind limbs [3,64] are listed as the most frequently affected body site. In a cohort of dogs aged 1–16 years, Smiech et al. (2019) found that Labrador Retrievers, French Bulldogs, and American Staffordshire Terriers were much more likely to develop MCTs on the trunk compared to other breeds [13]. This contrasts with our findings, as the French Bulldogs showed significantly fewer MCTs on the trunk than crossbreeds in our cohort.

Contrary to the findings of Kim et al. (2022), who analysed only dogs ≤ 1 year old [17], the trunk is still the most frequently affected site in dogs ≤ 1 year old in our group (29.4%). However, the authors did not specify whether both subcutaneous and cutaneous MCTs were analysed [17], limiting comparability to the present study. Nonetheless, we observed a significantly lower prevalence of MCTs on the trunk in this age group compared with the two- and three-year-olds in our group (trunk: 41.4%, *p* = 0.03).

The association between scrotal MCTs and French Bulldogs has not been reported before. The prevalence of scrotal MCTs of 1.3% is lower than in two other studies on dogs of all ages (4.0% [62] and 4.2% [83]). Furthermore, the prevalence of grade III in scrotal MCTs was lower than in a study including dogs between 1 and 16 years, which reports that 12 out of 21 scrotal MCTs (57%) were high grade [1,62]. Another study of a general dog population reports the prevalence of grade III MCTs at 19% [1]. Currently, the prognosis and biological behaviour of scrotal MCTs are controversially discussed [6,8]. Despite these findings on all-age dog populations, no significant differences between the proportion of grades of scrotal MCTs were found in the group of younger dogs of the present study.

The pinna, however, showed a significantly higher proportion of Patnaik grade I MCTs in the present study. Contrasting this are two studies on older dogs that report 50% Kiupel high grade in MCTs located on the pinna. However, since the extirpation of the sentinel lymph node (SNL) was a primary inclusion criterion for the study cohort [84], a selection bias for biologically aggressive MCTs is obvious, limiting comparability. A study of 28 pinnal MCTs from dogs of all ages found a 57% grade II and 29% grade III distribution. For grade I and grade II a very low rate of local recurrence was found and mean survival time was not reached, while grade III MCTs showed significantly lower survival times [85]. The data were collected from dogs treated at the veterinary clinic of the University of Pennsylvania and comparability may be impaired due to the inconsistent composition of that study’s and our cohorts.

Based on findings in the young dogs, the inclusion of survival data from pinnal MCTs may be of interest. Further analysis of clinical outcomes is required to clarify the prognosis of pinnal MCTs in young dogs.

### 4.5. Multiplicity

According to the amended staging criteria by Willmann et al. (2021), the occurrence of ≥3 cutaneous MCTs should be called multiple, and these dogs are to be sorted into stage II [86]. More than one MCT was submitted in 6% (*n* = 51) of the young dogs in the present study. Therefore, according to the original WHO system for clinical staging, 6% of our cohort presented with stage III MCTs [87]. Given the assumption that veterinarians excised all cutaneous lumps in dogs with multilocalised MCTs, only 1.2% of our cohort (*n* = 11) had multiple/stage II lesions according to the amended version of WHO criteria [86].

To the best of our knowledge, no reports of the prevalence of multiple MCTs in young dogs are available in the literature. In dogs of all ages, the prevalence of MCT multiplicity ranges from 6% to 21% [2,3,22,39]. The highest reported multiplicity rate was 56% (14/25 dogs) in an analysis of the University of Minnesota Veterinary Diagnostic Laboratory that included Pugs aged 3–12 years [4]. In contrast, 6/28 young Pugs (21.4%) showed multiple MCTs in the present study.

In our cohort, 14.2% of 88 Golden Retrievers had multiple MCTs, which was significantly more than in crossbreeds (4.4%). For Golden Retrievers, the rate of multiple occurrences was as high as 44% in an analysis of MCTs submitted to the Diagnostic Pathology Service of the Animal Health Trust in the US [88]. While multiplicity in the present study was defined as the simultaneous submission of MCTs, Murphy et al. (2006) [88] reported additive multiplicity over the course of one year. Thus, comparability is limited.

The dog with the highest number of multilocalised cutaneous MCTs in our cohort was a three-year-old male Weimaraner presenting with six MCTs located on the hind limb (*n* = 2), the fore limb, the pinna, the trunk, and the prepuce (one each). In general, the Weimaraner has been reported to get multiple MCTs [20,88], and showed the highest average number per patient in another study (3.6 per patient) [20].

As in previous studies on all-age dog populations [2,88], no statistical association between multiplicity and age, sex, histological grade, or tumour size was observed in the young dogs of the present study. Our findings indicate that, in young dogs, the multiplicity of MCTs is common in certain breeds. Increased vigilance for multilocalised MCTs may, therefore, be warranted from an early age in predisposed breeds.

### 4.6. Tumour Diameters

The diameter of canine cutaneous MCTs is associated with histologic grade [89,90] and may, therefore, be clinically relevant. A tumour diameter > 1.5 cm can be a predictor for high-grade malignancy in feline MCTs in cats, and the sensitivity is 80% [39]. In interpreting MCT size, variable degrees of interstitial oedema need to be considered [91].

In the present study, equivalent spherical diameters were analysed and derived from the volume of a rotational ellipsoid. The equivalent spherical tumour diameter in the present study ranged from 1.0 to 62 mm, and the median was 9.8 mm. Compared to median tumour diameters of all-age studies in the literature (20 mm [2], 21 mm [28], and 42 mm [3]), it was smaller.

Although a significant correlation between tumour size and histologic grade was observed, the Spearman rank correlation coefficient was low, indicating only a weak association. Therefore, tumour size alone may have limited value for predicting the histologic grade of cutaneous MCTs in young dogs. Generally, however, these findings are similar to an all-age dog population described by Itoh et al. (2014), where histologic grade was significantly associated with increased tumour size [90].

We found a significantly higher proportion of grade I MCTs in Pugs, who also had significantly smaller MCTs ($\tilde{x}$ = 5.0 mm) compared to crossbreeds ($\tilde{x}$ = 9.2 mm). Similarly, the largest median tumour diameter recorded in our group was in Shar Peis ($\tilde{x}$ = 30.9 mm).

In the literature, the size of MCTs has repeatedly been associated with survival time. One study of 73 dogs presenting with cutaneous MCTs ≥ 30 mm in diameter had a lower mean survival time than dogs with MCTs < 30 mm and a higher tendency towards lymph node metastasis [2]. Mullins et al. (2006) found that MCTs with a size ≥ 30 mm had a negative impact on survival time [40]. Another study reports that dogs with MCTs ≥ 50 mm have significantly shorter disease-free intervals after surgery (i.e., metastasis and/or recurrence) [90]. In the present study, 16 dogs presented with MCTs ≥ 30 mm (2/16 grade III and 1 grade I MCT), and 2 had MCTs ≥ 50 mm (1 grade II and 1 grade III). Further clinical analysis is required to determine whether survival times in larger MCTs are significantly different from those in smaller MCTs in young dogs.

### 4.7. KIT and Ki-67 Immunohistochemistry

Reports on KIT and Ki-67 immunoreactivity were available for 87 of 849 dogs. The dogs included in the analysis of KIT and Ki-67 are shown in full detail in Table S3 and were not preselected by the authors. The group reflects dogs for which immunohistochemical reports were available, based on order by the submitting veterinarian.

In the literature, no large-scale immunohistochemical analysis of cutaneous MCTs in young dogs has been conducted. In the present study, 22.9% of MCTs had more than 23 Ki-67-positive mast cells in a 10 × 10 mm grid area. In a group of 12 dogs ≤ 1 year of age, Rigas et al. (2020) [16] reported that 8/9 evaluated cutaneous MCTs exceeded the reference Ki-67 cut-off. Lymph node metastasis was found in 4/9, and 7/9 dogs were positive for the Exon 11 ITD. The breeds were heterogeneous, including one Shar Pei [16]. Whether an increased Ki-67 count translates into worse prognosis in young dogs has been disputed by Rigas et al. (2020), who reported all dogs to be alive and MCT-free after a median of 1115 days after diagnosis (3 years), despite an increased Ki-67 count in 8/9 cases [16]. Whether the high Ki-67 count can be attributed to the young age of the dogs rather than to tumour malignancy, as the authors suggest, remains speculative. No association between age and Ki-67 was noticed. However, the present study is not comparable due to the biased composition of included cases, based solely on veterinarians’ requests. An analysis of Ki-67 revealed that the Ki-67 labelling index increased with age [89], but survival data were not included.

KIT staining pattern I was found in 42.5% of cases (*n* = 38), and 54% of MCTs exhibited pattern II (*n* = 47). Pattern III was found in only 2 MCTs (2.3%). Of the KIT pattern I group, 81.1% had equal to or fewer than 23 Ki-67-positive cells in a 10 × 10 mm grid area, and 18.9% had more than 23. In the KIT staining pattern II group, 66% were below the cut-off, and 34% were above it. All of the KIT staining pattern III MCTs (*n* = 2) were below the Ki-67 cut-off. The lower rate of pattern III in our analysis is most likely due to the lack of Kiupel high-grade and Patnaik grade III MCTs in our KIT/Ki-67 cohort, where pattern III is more common [92]. To evaluate the prognostic significance of KIT and Ki-67 in young dogs, further studies that include survival data are required.

## 5. Conclusions

Cutaneous mast cell tumours may develop in young dogs, and, in summary, breed-specific attributes of cutaneous MCTs in the overall dog population were well reflected in our group of young dogs. Breeds with previously reported predisposition for cutaneous MCTs were represented, and the Danish–Swedish farm dog, English Setter, and Shih Tzu exhibited higher odds ratios for cutaneous MCTs than crossbreeds. For the breeds found, a higher risk might be considered and thorough diagnostic work-up of cutaneous lesions may be advisable even at an early age.

Compared with the general dog population, a lower proportion of Kiupel high-grade and Patnaik grade III tumours was observed. However, individual cases were histologically highly malignant. Significant differences in grade proportions were observed between breeds, underscoring the importance of histologic grading and breed-specific diagnostic approaches in young dogs. As in older dogs, cutaneous MCTs may be multilocalised, and the prevalence of multilocalised MCTs in the present study varied significantly by breed. Young dogs developed MCTs in similar locations to those in the overall dog population. However, MCTs on the pinna may require further attention regarding clinical prognosis. Immunohistochemical analysis revealed a predominance of KIT pattern II and Ki-67 below the proposed threshold in a subset of included dogs.

Further analysis and large-scale comparisons of survival data across standardised age groups are required to determine whether the findings translate into a clinical setting. Analysis of *kit* mutations in young dogs may be of interest for future studies.

## Figures and Tables

**Table 1:** Prevalence of Ki-67 and KIT results in young dogs. Percentages counted row-wise.

KIT Staining Pattern ^1^	Ki-67 ≤ 23; *n,* (%) ^2^	Ki-67 > 23; *n,* (%) ^2^
Pattern I (*n* = 38)	32 (81.1%)	6 (18.9%)
Pattern II (*n* = 47)	33 (66%)	14 (34%)
Pattern III (*n* = 2)	2 (100%)	0 (0%)
Total (*n* = 87)	67 (77.1%)	20 (22.9%)

^1^ KIT staining pattern, Kiupel et al. (2004) [23]. ^2^ Positive mast cells in 10 × 10 mm grid area, Webster et al. (2007) [61].

## Data Availability

The original contributions presented in the study are included in the article. Further inquiries can be directed to the corresponding authors.

## Supplementary Materials

The following supporting information can be downloaded at: https://www.mdpi.com/article/10.3390/vetsci13050500/s1. Table S1: Signalment of dogs and description of grades, anatomical sites, and multiplicity sorted by prevalence. Table S2: Breeds affected with cutaneous MCT sorted by prevalence. Table S3: Signalment data, grade, anatomic site, and tumour diameter of MCTs with immunohistochemistry (KIT and Ki-67). Table S4: Descriptive data of dogs with documented lymph node metastasis. Table S5: Odds ratios (ORs) and confidence intervals (CIs) for cutaneous mast cell tumours by breed (outcome: cutaneous MCTs/other) in dogs ≤ 3 years of age.
