# Supplementary material for: The Characteristics of 924 Cutaneous Mast Cell Tumours in Dogs ≤ 3 Years of Age—With a Short Literature Review of Feline, Equine, and Human Proliferative Mast Cell Disorders
Source: Vet Sci. 2026 May 20;13(5):500. doi: 10.3390/vetsci13050500 (PMC13211724; doi:10.3390/vetsci13050500)
Supplement: Supplementary file 1 [file vetsci-13-00500-s001.zip › vetsci-4283931-supplementary.pdf]

Table S1. Signalment of dogs and description of grades, anatomical sites, and multiplicity sorted by prevalence.

**Gender of dogs (∑ = 849)**	**Number**	**Percentage**
Male intact	298	35.1
Male neutered	117	13.8
Female intact	275	32.4
Female spayed	159	18.7
**Age of dogs (years, ∑ = 849)**		
≤1	149	17.9
> 1 ≤ 2	247	29.1
>2 ≤ 3	453	53.4
**Patnaik grades (∑ = 924)**		
Grade I	330	35.7
Grade II	580	62.8
Grade III	14	1.5
**Kiupel grades (∑ = 924)**		
Low-grade	903	97.6
High-grade	21	2.4
**Anatomical sites (∑ = 715)**		
Trunk	281	39.2
Hind limb	178	24.9
Fore limb	53	7.4
Head	72	10.1
Neck	30	4.2
Pinna	36	5
Digit_NOS	11	1.3
Scrotum	9	1.1
Tail	9	1.1
Multiple sites affected	34	4.0
**Multiplicity (∑ = 849)**		
Solitary MCT	788	94.0
Multilocalized MCT	51	6.0

Table S2. Breeds affected with cutaneous MCT sorted by prevalence.

Breed	Number	Percentage
Crossbreed	160	18,9%
Labrador Retriever	112	13,2%
French Bulldog	98	11,6%
Golden Retriever	88	10,4%
Boxer	75	8,8%
Pug	28	3,3%
Rhodesian Ridgeback	24	2,8%
Bernese Mountain Dog	15	1,8%
American Staffordshire Bullterrier	12	1,4%
Weimaraner	11	1,3%
Shih Tzu	10	1,2%
Great Swiss Mountain Dog	10	1,2%
Olde English Bulldog	10	1,2%
Shar Pei	10	1,2%
Maltese	9	1,1%
English Setter	8	0,9%
Dachshund	7	0,8%
American Bulldog	7	0,8%
Jack Russel Terrier	7	0,8%
Danish-Swedish farm dog	6	0,7%
Beagle	5	0,6%
Boston Terrier	5	0,6%
Bullmastiff	5	0,6%
Great Dane	5	0,6%
Flat Coated Retriever	5	0,6%
Staffordshire Bull Terrier	5	0,6%
Bordeaux Dog	4	0,5%
Cane Corso Italiano	4	0,5%
Doberman	4	0,5%
Dogo Argentino	4	0,5%
Shetland Sheepdog	4	0,5%
Yorkshire Terrier	4	0,5%
American Pitbull Terrier	3	0,4%
Chihuahua	3	0,4%
English Bulldog	3	0,4%
Magyar Vizsla	3	0,4%
Miniature Pinscher	3	0,4%
Australian Shepherd	2	0,2%
Belgian Shepherd Dog	2	0,2%
Bichon Frisé	2	0,2%
Border Collie	2	0,2%
Brabanter Griffon	2	0,2%
Bulldog	2	0,2%
Cairn Terrier	2	0,2%
Cocker Spaniel	2	0,2%
Continental Bulldog	2	0,2%
German Wirehair	2	0,2%
Fox Terrier	2	0,2%
Hovawart	2	0,2%
Kangal Shepherd Dog	2	0,2%
Kooikerhondje	2	0,2%
Münsterländer	2	0,2%
Nova Scotia Duck Tolling Retriever	2	0,2%
Podenco	2	0,2%
Poodle	2	0,2%
Welsh Corgi	2	0,2%

Continued below

Table S2. Breeds affected with cutaneous MCT sorted by prevalence. (Continuation)

Breed	Number	Percentage
Akita	1	0,1%
Alaskan Malamute	1	0,1%
Azawakh	1	0,1%
Beauceron	1	0,1%
Boxer	1	0,1%
Bretonic Spaniel	1	0,1%
Bull Terrier	1	0,1%
Chesapeak Bay Retriever	1	0,1%
Coton de Tuléar	1	0,1%
German Shepherd Dog	1	0,1%
English Pointer	1	0,1%
Husky	1	0,1%
Segugio Italiano	1	0,1%
Italian Greyhound	1	0,1%
Karelian Bear Hound	1	0,1%
Kaukasian Shepherd Dog	1	0,1%
Small Münsterländer	1	0,1%
Papillon	1	0,1%
Lhasa Apso	1	0,1%
Majorcan Mastiff	1	0,1%
Miniature Bull Terrier	1	0,1%
Norfolk Terrier	1	0,1%
Pinscher	1	0,1%
Portuguese Water Dog	1	0,1%
Retriever	1	0,1%
Setter	1	0,1%
Water Dog	1	0,1%
Miniature Poodle	1	0,1%
Miniature Schnauzer	1	0,1%
St. Bernhard	1	0,1%
Total	849	100%

Table S3. Signalment data, grade, anatomic site, and tumour diameter of MCTs with immunohistochemistry performed (KIT, Ki-67) staining.

ID	Gender	Breed	Age (years)	Tumour diameter (mm)	Patnaik grade	Anatomic site	KIT pattern	Ki-67 count
1	female intact	Golden Retriever	3	n.a.	II	Trunk	I	≤ 23
2	female intact	Labrador Retriever	1	n.a.	II	Trunk	I	≤ 23
3	female spayed	Pug	1	4,99	I	Trunk	I	≤ 23
4	male neutered	Crossbreed	3	5,94	II	Trunk	I	≤ 23
5	male intact	French Bulldog	2	n.a.	II	Hind limb	I	> 23
6	male intact	Great Swiss Mountain Dog	3	20,80	II	Hind limb	I	≤ 23
7	male intact	Labrador Retriever	2	n.a.	II	Hind limb	I	> 23
8	female spayed	Labrador Retriever	2	18,28	II	Hind limb	I	≤ 23
9	male neutered	Crossbreed	2	n.a.	I	Hind limb	I	≤ 23
10	male intact	Beauceron	1	14,78	I	n.a.	I	> 23
11	male intact	Bull Terrier	2	8,99	I	n.a.	I	≤ 23
12	female intact	Dogo Argentino	3	n.a.	II	n.a.	I	≤ 23
13	male intact	French Bulldog	3	n.a.	II	n.a.	I	≤ 23
14	male intact	French Bulldog	1	10,80	II	n.a.	I	≤ 23
15	female spayed	French Bulldog	3	n.a.	II	n.a.	I	≤ 23
16	female intact	Golden Retriever	3	n.a.	II	n.a.	I	≤ 23
17	female spayed	Golden Retriever	1	18,84	II	n.a.	I	≤ 23
18	male intact	Great Swiss Mountain Dog	3	15,53	II	n.a.	I	≤ 23
19	male intact	Münsterländer	3	n.a.	I	n.a.	I	≤ 23
20	female intact	Crossbreed	1	7,01	II	n.a.	I	≤ 23
21	female spayed	Crossbreed	3	13,64	II	n.a.	I	≤ 23
22	male intact	Akita	1	n.a.	II	Head	I	> 23
23	male neutered	Golden Retriever	2	5,94	II	Head	I	≤ 23
24	male intact	Maltese	1	n.a.	II	Head	I	≤ 23
25	female intact	Rhodesian Ridgeback	1	n.a.	II	Head	I	≤ 23
26	male neutered	Crossbreed	2	8,02	II	Head	I	≤ 23
27	male neutered	Crossbreed	1	n.a.	II	Head	I	≤ 23
28	female intact	Boxer	3	19,65	II	Fore limb	I	≤ 23
29	male intact	Golden Retriever	3	n.a.	II	Fore limb	I	> 23
30	female intact	Great Swiss Mountain Dog	3	8,74	I	Pinna	I	≤ 23
31	male intact	Golden Retriever	3	10,35	II	Neck	I	≤ 23
32	female intact	Golden Retriever	2	n.a.	I	Neck	I	≤ 23
33	male intact	Weimaraner	3	n.a.	II	Neck	I	≤ 23
34	male neutered	Crossbreed	2	n.a.	II	Neck	I	> 23
35	male neutered	Labrador Retriever	2	9,44	I	Tail	I	≤ 23
36	female spayed	Labrador Retriever	3	n.a.	II	Tail	I	≤ 23
37	male neutered	Crossbreed	2	n.a.	II	Digit	I	≤ 23
38	male intact	American Bulldog	2	12,15	II	Trunk	II	≤ 23
39	male intact	Boxer	2	2,12	II	Trunk	II	≤ 23
40	male intact	French Bulldog	3	9,85	II	Trunk	II	≤ 23
41	male intact	French Bulldog	3	n.a.	II	Trunk	II	> 23
42	female spayed	French Bulldog	3	n.a.	II	Trunk	II	> 23
43	male intact	Golden Retriever	1	n.a.	II	Trunk	II	≤ 23
44	male intact	Labrador Retriever	2	7,60	II	Trunk	II	≤ 23
45	female intact	Labrador Retriever	3	24,00	II	Trunk	II	> 23
46	female spayed	Labrador Retriever	2	n.a.	I	Trunk	II	≤ 23
47	male intact	Staffordshire Bull Terrier	3	n.a.	II	Trunk	II	≤ 23
48	female intact	Weimaraner	2	8,40	II	Trunk	II	≤ 23
49	male intact	Crossbreed	1	n.a.	II	Trunk	II	≤ 23
50	female intact	Crossbreed	3	1,00	II	Trunk	II	> 23
51	female intact	Crossbreed	2	n.a.	II	Trunk	II	> 23

Continuation below

Table S3. Signalment data, grade, anatomic site, and tumour diameter of MCTs with immunohistochemistry performed (KIT, Ki-67) staining. (Continuation)

ID	Gender	Breed	Age (years)	Tumour diameter (mm)	Patnaik grade	Anatomic site	KIT pattern	Ki-67 count
52	female spayed	Crossbreed	1	16,79	II	Trunk	II	≤ 23
53	female intact	American Bulldog	1	3,68	II	Hind limb	II	> 23
54	male intact	Boxer	3	17,75	II	Hind limb	II	≤ 23
55	male intact	French Bulldog	3	n.a.	II	Hind limb	II	> 23
56	male intact	French Bulldog	3	n.a.	II	Hind limb	II	> 23
57	female spayed	Golden Retriever	3	32,50	II	Hind limb	II	≤ 23
58	female intact	Labrador Retriever	2	n.a.	II	Hind limb	II	≤ 23
59	female intact	Labrador Retriever	3	25,71	II	Hind limb	II	≤ 23
60	female intact	Crossbreed	3	n.a.	II	Hind limb	II	≤ 23
61	male intact	Dachshund	3	n.a.	II	n.a.	II	≤ 23
62	male intact	Boxer	3	4,77	II	n.a.	II	≤ 23
63	male intact	Fox Terrier	1	n.a.	II	n.a.	II	≤ 23
64	male neutered	French Bulldog	2	n.a.	II	n.a.	II	> 23
65	male intact	Golden Retriever	3	n.a.	II	n.a.	II	> 23
66	female intact	Golden Retriever	2	8,31	II	n.a.	II	≤ 23
67	female intact	Labrador Retriever	2	5,64	I	n.a.	II	≤ 23
68	female spayed	Labrador Retriever	3	n.a.	II	n.a.	II	> 23
69	male intact	Crossbreed	3	23,13	II	n.a.	II	≤ 23
70	male neutered	Crossbreed	3	n.a.	II	n.a.	II	≤ 23
71	female intact	Crossbreed	1	n.a.	II	n.a.	II	> 23
72	female spayed	Crossbreed	2	5,76	II	n.a.	II	≤ 23
73	male intact	Kangal Shepherd Dog	2	n.a.	II	Head	II	> 23
74	male neutered	Rhodesian Ridgeback	2	n.a.	I	Head	II	≤ 23
75	male intact	Crossbreed	1	n.a.	II	Head	II	> 23
76	male intact	Golden Retriever	1	n.a.	II	Fore limb	II	≤ 23
77	male intact	Boxer	2	n.a.	II	Pinna	II	≤ 23
78	female intact	Boxer	3	9,14	I	Pinna	II	≤ 23
79	female intact	French Bulldog	2	n.a.	II	Pinna	II	≤ 23
80	female intact	Golden Retriever	3	18,09	II	Pinna	II	≤ 23
81	female intact	Great Swiss Mountain Dog	3	n.a.	II	Neck	II	≤ 23
82	female intact	Labrador Retriever	3	22,41	II	Neck	II	≤ 23
83	female intact	Crossbreed	3	n.a.	II	Neck	II	≤ 23
84	female intact	Golden Retriever	1	n.a.	II	Digit	II	> 23
85	female intact	Golden Retriever	3	8,83	II	Head	III	≤ 23
86	male intact	Golden Retriever	1	n.a.	I	Pinna	III	≤ 23
87	female intact	Crossbreed	3	n.a.	II	n.a.	II	≤ 23

Table S4. Descriptive data of dogs with documented lymph node metastasis.

ID	Breed	Age	Gender	Histologic Grade	Anatomic site
1	French Bulldog	1	f	III	Trunk
2	Hovawart	1	fs	III	Trunk
3	Crossbreed	3	mn	II	Trunk
4	Kooikerhondje	3	fs	III	Trunk
5	Labrador R	2	fs	II	Trunk

Table S5. Odds ratios (ORs) and confidence intervals for cutaneous mast cell tumours by breed (outcome: cutaneous MCTs/other) in dogs ≤ 3 years of age.

ID	Breed	Odds ratio	Lower 95% CI	Upper 95% CI	p-value
1	Danish-Swedish farm dog	8.11	3.33	19.77	< 0.0001
2	English Setter	6.68	3.20	13.97	< 0.0001
3	Shar Pei	5.50	2.76	10.94	< 0.0001
4	Golden Retriever	4.89	3.73	6.40	< 0.0001
5	German Boxer	4.15	3.13	5.51	< 0.0001
6	Great Swiss Mountain Dog	3.81	1.94	7.47	< 0.0001
7	Bullmastiff	3.38	1.33	8.57	0.03
8	Pug	3.29	2.15	5.04	< 0.0001
9	Labrador Retriever	2.82	2.20	3.60	< 0.0001
10	Weimaraner	2.71	1.43	5.12	0.006
11	Shih Tzu	2.30	1.19	4.46	0.04
12	French Bulldog	2.01	1.55	2.60	< 0.0001
13	Rhodesian Ridgeback	1.90	1.22	2.95	0.013
14	German Shepherd Dog	0.05	0.01	0.36	0.009
